# Scientific Items

**Published:** 1880-04-20

**Authors:** 


### SCIENTIFIC ITEMS.

**Paper Pulp From Poplar Wood.**—The Worcester "Spy" says:

It surprises people to see the great logs of poplar wood go through the powerful machine at the Connecticut river pulp mill at Holyoke. The wood as it is brought to the mill is about the size of cord wood used for fuel, and in this shape the machine takes it and gnaws it up very fine. So rapidly does this process go that the machine eats about seven and a half cords of wood a day, and this makes between three and four tons of pulp.

After coming from the machine the wood is put into vats and reduced by the action of chemicals. It is used for the manufacture of news and book paper, and pulp made from spruce wood, which has more fibre than poplar, is sometimes used in the cheaper grades of writing paper. Spruce is harder to reduce to pulp than poplar, and but little of it is used. The poplar trees in this vicinity have long since given out, and the wood is now mostly brought from Canada.—*Gaillard's Med. Journal.*

**Ophthalmoscopic Discovery.**—According to a foreign medical journal, one of the most interesting discoveries, of late, in physiology, is that made by Ball, namely, that the retina of the eye possesses in health a peculiar red color, which is constantly being destroyed by the influence of light, and as constantly being regenerated by the ordinary process of nutrition. The "vision red" or "erythopsin," as the discoverer names it, attains its maximum, after a night's rest and sleep, or when an animal has been kept for some hours in darkness; it is soluble in solutions of the biliary acids and of glycerine, and probably plays a part in the production of the red reflection from the fundus of the eye, seen on ophthalmoscopic examination.—*Journal of Materia Medica.*

**Valuable Sanitary Inventions.**—One of the most gratifying evidences of the progress of sanitary science is the useful inventions it is calling forth. One of the most valuable of these is the Eagle Odorless Apparatus for emptying privy-vaults and cess-pools without offence and without danger to health, the contents being transferred by an air-tight pump to air-tight barrels. The process is as economical as it is convenient and inoffensive, and needs only to be known to be universally adopted.

The air-tight garbage barrels made by the same company are equally to be commended as a convenient and cleanly substitute for that domestic nuisance, the swill-tub.—*Journal of Chemistry.*

A horse-stealing case tried at Lambeth recently revealed the little-known fact that horse-stealers commonly carry a stick of silver nitrate to darken white spots on horses to prevent identification.—*Boston Journal of Chemistry.*

**Remarkable Behavior of Silver Oxide.**—If two measured parts of silver oxide, perfectly dry, are rubbed in a porcelain mortar with one part antimony sulphide, the mixture readily takes fire. The same phenomenon occurs if silver oxide is ground along with amorphous phosphorus. If a drop of phenol is thrown upon silver oxide the latter is partially reduced, with the projection of sparks.—*Drug. Circular.*

**Atoms.**—The blow-pipe, although it had been for centuries employed by jewelers and others in soldering small objects, does not appear to have been brought into use for scientific purposes until about a hundred and fifty years ago, when Antony Swab, a Swede, utilized it in the examination of ores and minerals, for which it is invaluable and of the widest application.

**Tides in Mines.**—During the first six months of the present year, regular tides have been observed in the subterranean waters of the Fortschritt mine in Bohemia. This strange phenomenon has attracted the attention of the Academies of Science of Berlin and Vienna, but as yet no adequate explanation of it has been proposed.—*Drug. Circular.*

The number of telegrams received and sent by French offices rose from 3,600,000 in 1868, to over 11,000,000 in 1878, and last year it must have certainly exceeded 12,000,000. The French telegraphic network had in 1879 an extent of 113,669 kilometres, and at the end of December last year its extent was 171,500 kilometres.

Dr. Oliver Hoff, of San Francisco, who died recently, directed in his will that a monument, not to exceed $1000 in cost, should be placed over his grave, and forbade any society of which he was a member, or any friends, to pass resolutions of condolence over his decease, or communicate the fact to his friends in the East.

M. Fromme finds that Grove's battery is most efficacious when the nitric acid contains forty per cent. of water; the "strength" of Bunsen decreases with the concentration of the acid, and that of Daniell's increases with the concentration of sulphate of copper solution.

Prof. Erasmus Wilson, the dermatologist, who paid for bringing Cleopatra's needle to London, now proposes to erect, at his own cost, a swimming bath and chapel at Margate, England, to cost not less than $100,000.

An extraordinary prize of 3000 francs ($600) has been awarded by the French Academy of Sciences to Dr. Crookes, F. R. S., in recognition of his recent discoveries in molecular physics and radiant matter.

The Volta prize of 50,000 francs ($10,000) has been awarded to Professor Graham Bell by the committee appointed by the Minister of Public Instruction in France.

After careful examination, Dr. Treuman arrives at the conclusion that no process hitherto invented will keep iron effectively and durably from rust.

